# Polymorphism in lncRNA AC016683.6 and its interaction with smoking exposure on the susceptibility of lung cancer

**DOI:** 10.1186/s12935-018-0591-2

**Published:** 2018-07-04

**Authors:** Juan Li, Hang Li, Xiaoting Lv, Zitai Yang, Min Gao, Yanhong Bi, Ziwei Zhang, Shengli Wang, Zhigang Cui, Baosen Zhou, Zhihua Yin

**Affiliations:** 1 Department of Epidemiology, School of Public Health, China Medical University, Shenyang, 110122 People’s Republic of China; 2 Key Laboratory of Cancer Etiology and Intervention, University of Liaoning Province, No. 77 Puhe Road, Shenyang North New Area, Shenyang, 110122 People’s Republic of China; 3 School of Nursing, China Medical University, Shenyang, 110122 China

**Keywords:** Cancer, LncRNA, PAX8, Polymorphism, Susceptibility

## Abstract

**Background:**

Long non-coding RNAs play pivotal roles in the carcinogenesis of multiple types of cancers. This study is firstly to evaluate influence of rs4848320 and rs1110839 polymorphisms in long non-coding RNA AC016683.6 on the susceptibility of lung cancer.

**Methods:**

The present study was a hospital-based case–control study with 434 lung cancer patients and 593 cancer-free controls. Genotyping of the two SNPs detected by Taqman^®^ allelic discrimination method.

**Results:**

There were no statistically significant associations between rs4848320 and rs1110839 polymorphisms in AC016683.6 and risk of lung cancer in overall population. However, in the smoking population, rs4848320 and rs1110839 polymorphisms significantly increased the risk of lung cancer in dominant and homozygous models (Rs4848320: P = 0.029; Rs1110839: P = 0.034), respectively. In male population, rs1110839 genetic variant was related to the risk of lung cancer in all genetic models (GG vs. TT: P = 0.008; Dominant model: P = 0.029; Recessive model: P = 0.027) rather than heterozygous model. The crossover analyses provided rs4848320 and rs1110839 risk genotypes carriers combined with smoking exposure 2.218-fold, 1.755-fold increased risk of lung cancer (Rs4848320: P = 0.005; Rs1110839: P = 0.017). Additionally, there were significantly positive multiplicative interaction of rs4848320 polymorphism with smoking status, with adjusted OR of 2.244 (1.162–4.334), but rs1110839 polymorphism did not exist.

**Conclusions:**

Rs4848320 and rs1110839 polymorphisms may be associated with lung cancer susceptibility. Interaction of rs4848320 risk genotypes with smoking exposure may strengthen the risk effect on lung cancer.

## Background

Lung cancer is one of the most frequent malignant neoplasms in the world according to previous data gathered from different countries including China [1, 2]. The crude incidence and mortality of lung cancer were increasing rapidly over the past 30 years in China [3]. The occurrence and development of lung cancer may result from complicated interactions of environmental and genetic factors through previous epidemiological studies, which has considered tobacco exposure as a major environmental risk factor. With the in-depth exploration of genome-wide association studies (GWAS), multiple studies for underlying associations between single nucleotide polymorphisms (SNPs) in non-coding RNA genes and various cancers has gradually emerged with respect to genetic risk factors.

Long non-coding RNAs (lncRNAs), a kind of non-coding RNAs of more than 200 nucleotides in length, possesses the properties of lack transcriptional protein-coding function and open-reading frames [4–6]. On account of cellular function, lncRNAs could also be characteristically classified into tumor-suppressive and oncogenic lncRNAs in a way similar to the protein-coding genes [7]. LncRNAs may act as gene regulators through intricate mechanisms, including post-transcriptional, *trans*- and *cis*- gene regulation in carcinogenic pathways [8–11]. Most of previous studies have focused on revealing the critical role of lncRNAs for cancer risk, prognosis, diagnosis and targeted therapy, implying lncRNAs have shifted to the forefront of human cancer research.

Paired-box gene 8 (PAX8), one of members of the pair-box (PAX) gene family, is mapped on chromosome 2q13, which has multiple oncogenic properties such as inhibition of apoptosis to promote the survival of tumor cells, activation of BCl2 transcription and suppression of p53 [12–14]. A large number of studies have shown that PAX8 strikingly expressed in several types of tumors, exerts its biological functions and ultimately affects the development of cancer [15]. PAX2/5/8 could promote cell proliferation, but its transcription factors play conservative roles in affecting cell apoptosis [16–18]. Besides, specific protein–protein interactions could regulate the activity of PAX transcription factors. For example, activation of PAX8 transcriptional activity may be occur on condition that PAX2/5/8 interact with the tumor suppressor pRb [19]. Additionally, several studies had identified that the most common genetic event is the PAX8-PPARγ rearrangement except for the RAS gene mutations in follicular thyroid gland tumour [20–24]. PAX8-PPARγ rearrangement may affect the function of PPARγ protein and leads to impairment of normal PPARγ function eventually, which loss of the functions would result in uncontrolled cell growth [25, 26]. PAX8 may also bring about the elimination of the PTEN gene’s inhibitory effect by acting on the AKT to immunologically activate the PI3K signaling pathway [26]. Simultaneous activation of the signal pathways is highly carcinogenic, which may cause distant metastasis of tumour cells and the occurrence of locally infiltrating follicular carcinoma. Particularly, Kanteti et al. [27] reported that PAX8 is overexpressed in non-small cell lung cancer (NSCLC), whereas PAX5 protein is predominantly expressed in small cell lung cancer (SCLC) by analyzing the expression profile of the PAX gene family, and speculated that PAX8 is likely to interfere with p53 transcription and contribute to the development of tumor. Silencing of PAX8 expression may impair viability and motility of NSCLC cells in A549 cells [28]. Previously these studies had laid a solid foundation for gathering evidence to examine the hypothesis.

Recently, two studies has evaluated the association of expression quantitative trait loci (eQTL) SNPs in lncRNA AC016683.6, which located in the upstream region of PAX8 gene and could alter the expression of PAX8, with the susceptibility of cervical cancer [29] and the prognosis of hepatocellular carcinoma [30]. In this study, we hypothesized that rs4848320 and rs1110839 polymorphisms in AC016683.6 may shape the risk of lung cancer by supporting of the above evidence. To verify this hypothesis, we implemented a hospital-based case–control study consisting of 434 lung cancer cases and 593 cancer-free controls to evaluate the associations between the two SNPs and susceptibility of lung cancer. We also investigated the potential interaction of the two SNPs with smoking exposure on lung cancer risk, which was valuable for exploring cancer etiology and proposing improvement of environmental risk factors for cancer prevention.

## Materials and methods

### Study subjects and sample data collection

The hospital-based case–control study was conducted in Shenyang city of Liaoning Province, which located in the northeast China. All enrolled subjects were unrelated ethnic Han Chinese. Totally, 434 newly diagnosed lung cancer patients (111 males and 323 females) and 593 cancer-free subjects (180 males and 413 females) enrolled in the case and control group, respectively. The included criteria of the selected cases were as follows: (a) patients who examined through histopathological confirmation and newly diagnosed lung cancer; (b) The patients had no previous cancers; (c) These patients had not experienced radiotherapy or chemotherapy before. The included criteria of controls was consistent with the (b) (c) of the above criteria for cases. A small number of controls suffered mainly from coronary heart disease, hypertension and diabetes. Meanwhile, the recruited controls were from medical examination centers of the hospitals in the same period and matched to the selected patients on age (± 5 years) to ensure comparability among subjects. The study has acquired the approval of the Institutional Review Board of China Medical University. All enrolled participants or every participant’s representatives signed the informed consent according to relevant regulations. Each participants donated 10 ml peripheral blood and possess capability for undergoing a 1.5 h interview to complete the demographic data by filling out questionnaire. Additionally, a subject who smoked more than 100 cigarettes in the whole lifetime is considered as a smoker, otherwise, he or she is regarded as a non-smoker.

### SNP selection and genotyping

Based upon the data from Regulome Database, the study selected the rs4848320 and rs1110839 SNPs in PAX8-AS1 (http://www.regulomedb.org) [31], which are meet the criteria of minor allele frequency (MAF) > 0.05 in Han Chinese population [32, 33]. Following the previous method [34], we isolated genomic DNA samples from the vein blood of each participants by Phenol–chloroform extraction. Genotyping of the two SNPs conducted by using an Applied Biosystems 7500 FAST Real-Time PCR System (Foster City, CA, USA) utilizing Taqman^®^ allelic discrimination (Applied Biosystems, Foster City, CA) with commercial primer–probe set. The reaction condition of real-time PCR was set at 10 min for 95 °C, 30 s at 92 °C and 1 min at 60 °C for all 47 cycles.

### Statistical analysis

T test, χ^2^ test and logistic regression analysis tested the differences between the case and control group according to type of demographic variables (including categorical and continuous variables). A goodness-of-fit χ^2^ test was performed to estimate the Hardy–Weinberg equilibrium (HWE) of the control group. The linear-by-linear association of χ^2^ test was conducted to assess trend analysis in a similar dose-dependent manner. The odds ratios (ORs) and their 95% confidence intervals (CIs) were used to evaluate the relationships between rs4848320, rs1110839 genotypes and lung cancer risk by means of logistic regression analysis. Crossover analysis was to examine interaction between the SNPs and smoking exposure. The multiplicative interaction was evaluated by OR and its 95% CI with the unconditional logistic regression model. Assessment of the addictive interaction applied relative excess risk due to interaction (RERI), synergy index (S) and attributable proportion due to interaction (AP). If the 95% CI of RERI/AP did not contain 0 and the 95% CI of S did not contain 1, the addictive interaction was existed [35]. All statistical analyses was obtained by performing SPSS 22.0 software (IBM SPSS, Inc. Chicago, IL, USA), and P < 0.05 was regarded as a statistically significant result under conditions of the all tests with two-sided.

## Results

### Baseline characteristics

Table 1 clearly presented the demographic characteristics of 593 controls and 434 lung cancer patients, which covers 331 non-small cell lung cancer (NSCLC), 233 adenocarcinoma (AD), 89 squamous cell carcinoma (SQ) and 61 small cell lung cancer (SCLC), respectively. The age mean ± SD of case group and control group were 57.97 ± 11.85 and 56.67 ± 15.88, respectively. There were no statistical differences on age and gender of the case and control groups. However, the distribution of smoking status in both groups presented the significant difference (P = 0.044). Therefore, all the further statistical analyses adjusted by age, gender and smoking status. Genotype frequency distributions of the two SNPs in control group was in agreement with HWE (P = 0.556 for rs4848320, P = 0.643 for rs1110839), which indicated selected subjects were a randomly representative sample from the overall population.

**Table 1:** Demographics of patients with lung cancer and controls

Variable	Case (%) (434)	Control (%) (593)	P value
Age, year (mean ± SD)	57.97 ± 11.85	56.67 ± 15.88	0.134
Age, (year)			
≤ 57	190 (43.8)	264 (44.5)	0.813
> 57	244 (56.2)	329 (55.5)	
Gender			
Male	111 (25.6)	180 (30.4)	0.093
Female	323 (74.4)	413 (69.6)	
Smoking status			
Never	338 (77.9)	475 (80.1)	0.044^a^
Ever	96 (22.1)	118 (19.9)	
Clinical stage			
I, II	86 (19.8)		
III	198 (45.6)		
IV	59 (13.6)		
Other	91 (21.0)		
Histology			
AD	233 (53.6)		
SQ	89 (20.5)		
SCLC	61 (14.1)		
Other	51 (11.8)		

*AD* adenocarcinoma, *SQ* squamous cell carcinoma, *SCLC* small cell lung cancer

^a^ P value was adjusted for age, gender and smoking

### Genotype distribution and lung cancer susceptibility

Table 2 summarized the associations between the SNPs genotypes and the susceptibility to lung cancer as well as NSCLC. No significantly statistical significance was in all genetic (CT vs. CC: OR = 1.036, 95% CI = 0.788–1.362, P = 0.800; TT vs. CC: OR = 1.041, 95% CI = 0.482–2.247, P = 0.918; Dominant model: OR = 1.036, 95% CI = 0.795–1.352, P = 0. 0.792; Recessive model: OR = 1.030, 95% CI = 0.479–2.212, P = 0.940, adjusted by age, gender and smoking) models for rs4848320 polymorphism. Rs1110839 genetic variation was not significantly statistical significance in all (TG vs. TT: OR = 1.083, 95% CI = 0.830–1.413, P = 0.559; GG vs. TT: OR = 1.079, 95% CI = 0.714 –1.632, P = 0.717; Dominant model: OR = 1.082, 95% CI = 0.839–1.394, P = 0.543; Recessive model: OR = 1.034, 95% CI = 0.702–1.525, P = 0.864, adjusted by age, gender and smoking) models. Moreover, similar results occurred in NSCLC, lung adenocarcinoma and squamous cell carcinoma subgroups (Tables 2 and 3). Subsequently, we estimated the combined effect of variant alleles (Rs4848320-T and Rs1110839-G) on the risk of lung cancer and NSCLC (shown in Table 4). We obtained that lung cancer and NSCLC risks was not remarkable significance with the increasing number of risk genotypes of the rs4848320 and rs1110839 in a similar dose-dependent manner (LC: P = 0.893; NSCLC: P = 0.964). The results of stratified analyses by smoking exposure, gender and age presented in Tables 5, 6 and 7, respectively. In the smoking population, patients with CT and TT genotypes were significantly increased with the risk of lung cancer in dominant (Rs4848320: OR = 1.929, 95% CI = 1.070–3.479, P = 0.029, adjusted by age and gender) model rather than other three models. Rs110839 GG genotype was significantly associated with increased risk of lung cancer (OR = 3.224, 95% CI = 1.089–9.544, P = 0.034). In male population, rs1110839 genotypes were related to significantly increased risk of lung cancer in all genetic models (GG vs. TT: OR = 3.096, 95% CI = 1.343–7.134, P = 0.008; Dominant model: OR = 1.804, 95% CI = 1.061–3.067, P = 0.029; Recessive model: OR = 2.376, 95% CI = 1.102–5.121, P = 0.027) except for heterozygous model. However, the results of non-smoking, age and female subgroups did not reveal significant effect on lung cancer susceptibility.

**Table 2:** Association between the two SNPs and risk of lung cancer and non-small cell lung cancer

Genotype	Controls (%) (593)	Lung cancer			Non-small cell lung cancer		
		Cases (%) (434)	OR^a^ (95% CI)	P value	Cases (%)(331)	OR^a^ (95% CI)	P value
Rs4848320							
CC (ref)	402 (67.8)	291 (67.1)	1.00 (ref)		220 (66.5)	1.00 (ref)	
CT	175 (29.5)	131 (30.2)	1.036 (0.788–1.362)	0.800	101 (30.5)	1.160 (0.787–1.427)	0.703
TT	16 (2.7)	12 (2.8)	1.041 (0.482–2.247)	0.918	10 (3.0)	1.168 (0.515–2.647)	0.710
CT + TT vs CC			1.036 (0.795–1.352)	0.792		1.069 (0.801–1.426)	0.653
TT vs CT + CC			1.030 (0.479–2.212)	0.940		1.147 (0.509–2.586)	0.741
Rs1110839							
TT (ref)	250 (42.2)	174 (40.1)	1.00 (ref)		134 (40.5)	1.00 (ref)	
TG	274 (46.2)	209 (48.2)	1.083 (0.830–1.413)	0.559	158 (47.7)	1.068 (0.799–1.428)	0.656
GG	69 (11.6)	51 (11.8)	1.079 (0.714–1.632)	0.717	39 (11.8)	1.066 (0.679–1.672)	0.782
TG + GG vs TT			1.082 (0.839–1.394)	0.543		1.068 (0.810–1.407)	0.642
GG vs TG + TT			1.034 (0.702–1.525)	0.864		1.029 (0.674–1.570)	0.895

*SNP* single nucleotide polymorphism, *OR* odds ratio, *CI* confident interval

OR^a^ was adjusted by age, gender and smoking

**Table 3:** Association between the two SNPs and risk of lung adenocarcinoma and squamous cell lung cancer

Genotype	Controls (%) (593)	Lung adenocarcinoma			Squamous cell carcinoma		
		Cases (%) (233)	OR^a^ (95 %CI)	P value	Cases (%) (89)	OR^a^ (95% CI)	P value
Rs4848320							
CC (ref)	402 (67.8)	159 (68.2)	1.00 (ref)		58 (65.2)	1.00 (ref)	
CT	175 (29.5)	68 (29.2)	0.957 (0.679–1.350)	0.804	28 (31.5)	1.127 (0.690–1.840)	0.634
TT	16 (2.7)	6 (2.6)	0.910 (0.343–2.413)	0.849	3 (3.4)	1.230 (0.339–4.466)	0.753
CT + TT vs CC			0.953 (0.683–1.331)	0.779		1.135 (0.706–1.826)	0.600
TT vs CT + CC			0.922 (0.350–2.431)	0.870		1.184 (0.329–4.259)	0.796
Rs1110839							
TT (ref)	250 (42.2)	102 (43.8)	1.00 (ref)		30 (33.7)	1.00 (ref)	
TG	274 (46.2)	107 (45.9)	0.960 (0.691–1.334)	0.808	46 (51.7)	1.336 (0.813–2.195)	0.253
GG	69 (11.6)	24 (10.3)	0.805 (0.474–1.368)	0.423	13 (14.6)	1.605 (0.787–3.273)	0.193
TG + GG vs TT			0.928 (0.678–1.270)	0.639		1.388 (0.864–2.229)	0.175
GG vs TG + TT			0.822 (0.498–1.359)	0.445		1.362 (0.712–2.608)	0.350

*SNP* single nucleotide polymorphism, *OR* odds ratio, *CI* confident interval

OR^a^ was adjusted by age, gender and smoking

**Table 4:** Cumulative effects of rs4848320-T and rs1110839-G on lung cancer and non-small cell lung cancer susceptibility

Variant	Controls (%) (593)	Lung cancer			Non-small cell lung cancer		
		Cases (%) (434)	OR^a^ (95% CI)	P value	Cases (%) (331)	OR^a^ (95% CI)	P value
No. of alleles							
0 (ref)	232 (39.1)	168 (38.7)	1.00 (ref)		129 (39.0)	1.00 (ref)	
1	174 (29.3)	115 (26.5)	0.893 (0.654–1.218)	0.474	85 (25.7)	0.871 (0.619–1.225)	0.427
2	132 (22.3)	112 (25.8)	1.167 (0.846–1.612)	0.346	88 (26.6)	1.207 (0.852–1.710)	0.291
3–4	55 (9.3)	39 (9.0)	0.985 (0.622–1.560)	0.948	29 (8.8)	0.947 (0.572–1.569)	0.833
Trend			P^b^ = 0.893			P^b^ = 0.964	
0	232 (39.1)	168 (38.7)	1.00 (ref)		129 (39.0)	1.00 (ref)	
1–4	361 (60.9)	266 (61.3)	1.007 (0.780–1.301)	0.955	202 (61.0)	1.005 (0.761–1.328)	0.971

*OR* odds ratio, *CI* confident interval

OR^a^ was adjusted by age, gender and smoking

^b^ P value was calculated by linear-by-linear association of χ^2^ test

**Table 5:** Stratified analyses of association between the two SNPs and lung cancer risk by smoking exposure

SNP	Genotype	Smoking exposure	Controls (%)	Cases (%)	ORª (95% CI)	P value
Rs4848320	CC	Ever	88 (74.6)	58 (60.4)	1.00 (ref)	
	CT		29 (24.6)	32 (33.3)	1.695 (0.922–3.119)	0.090
	TT		1 (0.8)	6 (6.3)	8.219 (0.957–70.618)	0.055
	CT + TT vs CC				1.929 (1.070–3.479)	0.029
	TT vs CT + CC				7.048 (0.827–60.092)	0.074
	CC	Never	314 (66.1)	233 (68.9)	1.00 (ref)	
	CT		146 (30.7)	99 (29.3)	0.914 (0.671–1.245)	0.568
	TT		15 (3.2)	6 (1.8)	0.563 (0.213–1.484)	0.245
	CT + TT vs CC				0.882 (0.653–1.192)	0.415
	TT vs CT + CC				0.578 (0.220–1.519)	0.266
Rs1110839	TT	Ever	50 (42.4)	30 (31.3)	1.00 (ref)	
	TG		62 (52.5)	53 (55.2)	1.376 (0.763–2.482)	0.288
	GG		6 (5.1)	13 (13.5)	3.224 (1.089–9.544)	0.034
	TG + GG vs TT				1.539 (0.867–2.731)	0.141
	GG vs TG + TT				2.652 (0.954–7.371)	0.062
	TT	Never	200 (42.1)	144 (42.6)	1.00 (ref)	
	TG		212 (44.6)	156 (46.2)	1.010 (0.748–1.364)	0.950
	GG		63 (13.3)	38 (11.2)	0.847 (0.534–1.341)	0.478
	TG + GG vs TT				0.973 (0.731–1.294)	0.849
	GG vs TG + TT				0.842 (0.546–1.299)	0.437

*SNP* single nucleotide polymorphism, *OR* odds ratio, *CI* confident interval

OR^a^ was adjusted by age and gender

**Table 6:** Stratified analyses of association between the two SNPs and lung cancer risk by gender

SNP	Genotype	Gender	Controls (%)	Cases (%)	ORª (95% CI)	P value
Rs4848320	CC	Male	124 (68.9)	69 (62.2)	1.00 (ref)	
	CT		51 (28.3)	37 (33.3)	1.334 (0.774–2.298)	0.299
	TT		5 (2.8)	5 (4.5)	2.042 (0.500–8.333)	0.320
	CT + TT vs CC				1.389 (0.821–2.350)	0.220
	TT vs CT + CC				1.860 (0.461–7.498)	0.383
	CC	Female	278 (67.3)	222 (68.7)	1.00 (ref)	
	CT		124 (30.0)	94 (29.1)	0.959 (0.695–1.323)	0.797
	TT		11 (2.7)	7 (2.2)	0.824 (0.313–2.166)	0.694
	CT + TT vs CC				0.948 (0.693–1.297)	0.737
	TT vs CT + CC				0.835 (0.319–2.183)	0.713
Rs1110839	TT	Male	79 (43.9)	32 (28.8)	1.00 (ref)	
	TG		84 (46.7)	60 (54.1)	1.582 (0.908–2.757)	0.105
	GG		17 (9.4)	19 (17.1)	3.096 (1.343–7.134)	0.008
	TG + GG vs TT				1.804 (1.061–3.067)	0.029
	GG vs TG + TT				2.376 (1.102–5.121)	0.027
	TT	Female	171 (41.4)	142 (44.0)	1.00 (ref)	
	TG		190 (46.0)	149 (46.1)	0.957 (0.702–1.305)	0.783
	GG		52 (12.6)	32 (9.9)	0.751 (0.457–1.233)	0.258
	TG + GG vs TT				0.913 (0.680–1.228)	0.548
	GG vs TG + TT				0.768 (0.481–1.228)	0.271

*SNP* single nucleotide polymorphism, *OR* odds ratio, *CI* confident interval

OR^a^ was adjusted by age and smoking

**Table 7:** Stratified analyses of association between the two SNPs and lung cancer risk by age

SNP	Genotype	Age	Controls (%)	Cases (%)	ORª (95% CI)	P value
Rs4848320	CC	≤ 57	180 (68.2)	132 (69.5)	1.00 (ref)	
	CT		78 (29.5)	54 (28.4)	0.852 (0.548–1.324)	0.476
	TT		6 (2.3)	4 (2.1)	1.323 (0.319–5.496)	0.700
	CT + TT vs CC				0.876 (0.570–1.347)	0.547
	TT vs CT + CC				1.385 (0.336–5.716)	0.652
	CC	> 57	222 (67.5)	159 (65.2)	1.00 (ref)	
	CT		97 (29.5)	77 (31.6)	1.109 (0.768–1.602)	0.581
	TT		15 (3.0)	8 (3.3)	1.214 (0.462–3.189)	0.694
	CT + TT vs CC				1.119 (0.784–1.596)	0.537
	TT vs CT + CC				1.175 (0.451–3.066)	0.741
Rs1110839	TT	≤ 57	115 (43.6)	85 (44.7)	1.00 (ref)	
	TG		123 (46.6)	86 (45.3)	0.943 (0.621–1.434)	0.784
	GG		26 (9.8)	19 (10.0)	1.288 (0.633–2.620)	0.486
	TG + GG vs TT				0.994 (0.666–1.484)	0.976
	GG vs TG + TT				1.327 (0.674–2.612)	0.413
	TT	> 57	135 (40.1)	89 (36.5)	1.00 (ref)	
	TG		151 (45.9)	123 (50.4)	1.299 (0.902–1.872)	0.160
	GG		43 (13.1)	32 (13.1)	1.195 (0.698–2.048)	0.516
	TG + GG vs TT				1.276 (0.902–1.806)	0.169
	GG vs TG + TT				1.034 (0.628–1.703)	0.894

*SNP* single nucleotide polymorphism, *OR* odds ratio, *CI* confident interval

OR^a^ was adjusted by gender and smoking

### Interaction between SNPs and smoking exposure

Table 8 provided the results of the crossover analysis. TT and CT genotypes carriers with smoking exposure were significantly associated with 2.218-fold increased risk to lung cancer (P = 0.005) for rs4848320. Additionally, rs1110839 TG and GG genotypes carriers with smoking exposure 1.755-fold increased risk to lung cancer (P = 0.017). However, the parameters of additive interaction presented that rs4848320 and rs1110839 polymorphisms had no additive interaction with smoking exposure (shown in Table 9). The OR value of multiplicative interaction of rs4848320 risk genotypes with smoking exposure more than 1 indicated that the interaction was positive multiplicative interaction, with a P value of 0.016 (P < 0.05) (Table 10). In other words, when the rs4848320 TT and CT genotypes coexist with smoking exposure, the interaction between the two risk factors becomes stronger, and its biological significance is a synergistic effect.

**Table 8:** Crossover analysis of interaction between rs4848320, rs1110839 risk genotypes and smoking exposure

SNPs	Genotype	Smoking exposure	Controls (%)	Cases (%)	OR^a^ (95% CI)	P value
Rs4848320	CC	Never	314 (53.0)	233 (53.7)	1 (ref)	
	CT + TT	Never	161 (27.2)	105 (24.2)	0.875 (0.648–1.182)	0.384
	CC	Ever	88 (14.8)	58 (13.4)	1.129 (0.738–1.728)	0.576
	CT + TT	Ever	30 (5.1)	85 (31.8)	2.218 (1.272–3.869)	0.005
Rs1110839	TT	Never	200 (33.7)	144 (33.2)	1 (ref)	
	TG + GG	Never	275 (46.4)	194 (44.7)	0.968 (0.729–1.286)	0.822
	TT	Ever	50 (8.4)	30 (6.9)	1.043 (0.611–1.782)	0.877
	TG + GG	Ever	68 (11.5)	66 (15.2)	1.755 (1.108–2.781)	0.017

*CI* confidence interval, *OR* odds ratio

OR^a^ was adjusted by age, gender

**Table 9:** Addictive interaction between rs4848320, rs1110839 risk genotypes and smoking exposure

Measure	Rs4848320		Rs1110839	
	Estimate	95% CI	Estimate	95% CI
RERI	0.940	0.045–1.835	0.535	− 0.092–1.162
AP	0.551	0.231–0.870	0.397	− 0.007–0.801
S	− 3.306	#NUM!	− 1.862	#NUM!

*CI* confidence interval, *RERI* relative excess risk due to interaction, *AP* attributable proportion due to interaction, *S* synergy index

**Table 10:** Multiplicative interaction between rs4848320, rs1110839 risk genotypes and smoking exposure

SNPs	Variables	OR^a^ (95% CI)	P value
Rs4848320	Smoking exposure	0.503 (0.196–1.292)	0.153
	Rs4848320 CT + TT	0.875 (0.648–1.182)	0.384
	Interaction	2.244 (1.162–4.334)	0.016
Rs1110839	Smoking exposure	0.600 (0.202–1.783)	0.358
	Rs1110839 TG + GG	0.968 (0.729–1.286)	0.822
	Interaction	1.738 (0.920–3.285)	0.089

*CI* confidence interval, *OR* odds ratio

OR^a^ was adjusted by age, gender

## Discussion

The current case–control study initially investigated the associations between polymorphisms in AC016683.6 and lung cancer susceptibility. We obtained that rs4848320 and rs1110839 polymorphisms significantly increased the risk of lung cancer in the smoking and male population. Subsequently, the crossover analyses provided the two SNPs risk genotypes carriers with smoking exposure increased the risk of lung cancer. Moreover, the interaction between rs4848320 risk genotypes and smoking was positive multiplicative interaction.

Genome-wide cancer mutation analyses are uncovering how the particular properties of lncRNAs to contribute crucial cancer phenotypes through the regulation of lncRNAs transcription [6], suggesting that lncRNAs are powerful modulators, involving chromatin interaction, transcriptional regulation, and DNA, Protein, RNA processing [36]. McGinnis et al. initially discovered the paired box gene family in Drosophila [37]. Each member among the family is a very conservative transcriptional regulator and actively participates in the sophisticated regulation of intracellular signal transduction. The cooperative regulation among the PAX genes plays a crucial role in different biological processes such as the transcription of development-related genes, the promotion of cell proliferation and differentiation, the inhibition of apoptosis, the induction of directional metastasis of precursor cells as well as selective processing of different splice sites of the precursor mRNA [38–40]. PAX8, a product of the pair-box gene family, plays a complex role in the developmental of various disease including cancers [12]. Overwhelming evidence elucidated that PAX8 is a efficacious marker to distinguish serous ovarian tumor [41, 42], metastatic müllerian carcinomas [43], renal cell carcinoma [44]. The expression of PAX5 and PAX8 are different in lung cancer tissues in order to further identify potential therapeutic targets, while PAX5 inclined to expressed in SCLC cells, overexpression of PAX8 in most NSCLC cell lines [27]. Moreover, the results of Muratovska et al. presented that PAX genes influence cell survival in specific types of cancer [17].

Previous studies demonstrated that AC016683.6 eQTLs SNPs might contribute to susceptibility or prognosis to different cancers [29, 30, 45]. Han et al. firstly conducted a case–control study, which revealed that rs4848320 TT and rs1110839 GG decreased risk of cervical cancer, with the corresponding adjusted ORs (95% CIs) of 0.58 (0.36–0.93) and 0.75 (0.58–0.97), respectively [29]. However, another study found that rs4848320 polymorphism may also be a risk factor for the risk of childhood acute lymphoblastic leukemia [45]. Moreover, Ma et al. demonstrated the SNPs in AC016683.6 were significantly associated with the prognosis of hepatocellular carcinoma [30]. However, the study did not provide both rs4848320 and rs1110839 polymorphisms associated with lung cancer risk in overall population. We identified that rs4848320 and rs1110839 polymorphisms increased the risk of lung cancer in the smoking and male population. Besides, we founded that there was positive multiplicative interaction between rs4848320 risk genotypes and smoking exposure, indicating that the interaction increased the susceptibility to lung cancer.

Previous evidence identified that SNP mutations in lncRNAs may alter their structural stability, generate alternative splicing, impair the translation of target mRNAs, and ultimately affect the risk of various cancers [6]. For example, rs35592567 polymorphism affect the expression of TP63 by interfering miR-140, which may serve as a reasonable explanation for the increased susceptibility of gastric cancer [46]. Additionally, Xue et al. reported that rs7958904 G > C strikingly altered the secondary structure of HOTAIR, suggesting the SNPs affect the susceptibility to colorectal cancer. LncRNA AC016683.6 is located in the intron region of PAX8, which mapped on chromosome 2q13. Rs1110839 and rs4848320 polymorphisms in AC016683.6 may be the eQTLs SNPs for PAX8 gene. Consequently, it is likely that the two SNPs could influence the specific interaction between AC016683.6 and PAX8, directly regulating the expression of PAX8. Therefore, we need to explore the functional properties of SNPs in oncogenic lncRNAs, which may be beneficial for developing the potential of lncRNAs in the diagnosis and treatment of cancer.

The study should emphatically highlight several implicated limitations. Firstly, the subjects from Shenyang city may lack of representative to some extent. Secondly, sample size of the study is limited, especially stratified subgroup. Thirdly, functional verification of two SNPs in AC016683.6 did not in our current study. Therefore, more large-scale studies confirm these results of the study across different ethnicities in the future.

## Conclusions

Rs4848320 and rs1110839 genetic variants may be associated with the increased risk of lung cancer. The rs4848320 risk genotypes combined with smoking status strengthened the risk effect on lung cancer risk. However, the rs4848320 and rs1110839 genetic variants in AC016683.6 may serve as potential genetic risk factors for lung cancer susceptibility, which needs to be verified in a larger population. Additionally, biological function of the two SNPs in AC016683.6 need further to clarify in lung cancer. Previous studies and the current study provide novel clues for functional analysis of cancer-related susceptibility loci.

## Abbreviations

LncRNA: long non-coding RNA
PAX8: paired-box gene 8
SNP: single nucleotide polymorphism
LC: lung cancer
NSCLC: non-small cell lung cancer
AD: adenocarcinoma
SQ: squamous cell carcinoma
SCLC: small cell lung cancer
HWE: Hardy–Weinberg equilibrium
95% CI: 95% confidence interval
OR: odds ratio
RT-PCR: real-time polymerase chain reaction
